# Discovery of Novel Dual Adenosine A_2A_ and A_1_ Receptor Antagonists with 1*H*-Pyrazolo[3,4*-d*]pyrimidin-6-amine Core Scaffold as Anti-Parkinson’s Disease Agents

**DOI:** 10.3390/ph15080922

**Published:** 2022-07-25

**Authors:** Juyoung Jung, Yoonsuk Lee, An-Na Moon, Jihyae Ann, Jin Ju Jeong, Nayeon Do, Jeewoo Lee

**Affiliations:** 1 iLeadBMS Co., Ltd., Hwaseong-si 18469, Korea; julia.jung@ileadbms.com (J.J.); ivo.lee@ileadbms.com (Y.L.); anna.moon@ileadbms.com (A.-N.M.); 2 Laboratory of Medicinal Chemistry, College of Pharmacy, Seoul National University, Seoul 08826, Korea; jihuya@gmail.com (J.A.); lsys7169@naver.com (J.J.J.); nydoh@snu.ac.kr (N.D.)

**Keywords:** adenosine receptor antagonist, Parkinson’s disease, animal model, pharmacokinetic

## Abstract

New compounds with 1*H*-pyrazolo [3,4*-d*]pyrimidin-6-amine core scaffolds were synthesized and characterized in vitro to determine their affinity for human A_2A_ and A_1_ receptors. Among the tested compounds, a few compounds displayed nanomolar binding affinities for both receptors. One particular compound, **11o,** showed high binding activities (*h*A_2A_ K_i_ = 13.3 nM; *h*A_1_ K_i_ = 55 nM) and full antagonism (*h*A_2A_ IC_50_ = 136 nM; *h*A_1_ IC_50_ = 98.8 nM) toward both receptors. Further tests showed that **11o** has low hepatic clearance and good pharmacokinetic properties in mice, along with high bioavailability and a high brain plasma ratio. In addition, **11o** was associated with very low cardiovascular risk and mutagenic potential, and was well-tolerated in rats and dogs. When tested in an MPTP-induced mouse model of Parkinson’s disease, **11o** tended to improve behavior. Moreover, **11o** dose-dependently reversed haloperidol-induced catalepsy in female rats, with graded ED_50_ of between 3 and 10 mg/kg. Taken together, these results suggest that this potent dual A_2A_/A_1_ receptor antagonist, **11o**, is a good candidate for the treatment of Parkinson’s disease with an excellent metabolic and safety profile.

## 1. Introduction

Parkinson’s disease (PD) is the second most prevalent neurodegenerative disease worldwide, but still lacks a curative therapeutic treatment. Loss of dopaminergic neurons in the substantia nigra pars compacta, leading to striatal dopamine depletion, is the core pathophysiological finding in patients with PD. Currently, levodopa (L-DOPA) is the standard of care (SOC) to treat the motor symptoms of PD [1]. Levodopa alleviates the motor symptoms of patients with PD by replacing the depleted dopamine in the brain. However, PD symptoms become increasingly refractory to SOC as the disease progresses, resulting in motor complications, including motor-response oscillations (on-off periods) and drug-induced dyskinesias, probably due to discontinuous drug delivery and its short half-life [2]. Several FDA-approved therapies exist to treat off periods in patients with PD, including levodopa inhalation powder, entacapone tablets, carbidopa/levodopa enteral suspension, carbidopa/levodopa/entacapone tablets, apomorphine hydrochloride injection, safinamide tablets and istradefylline tablets. Despite these options, patients with PD still need therapies that are more efficacious, longer lasting and safer to use.

Adenosinergic modulation of the indirect striatal output pathway remains one of the most well-investigated nondopaminergic approaches to control PD symptoms. Adenosine affects the dopaminergic system via receptor interactions and intracellular signaling in pre- and postsynaptic neurons. Four known subtypes of adenosine receptors have been identified, A_1_, A_2A_, A_2B_ and A_3_, where the role of the A_2A_ receptor in PD is the most extensively studied and well-known.

**Figure 1:**
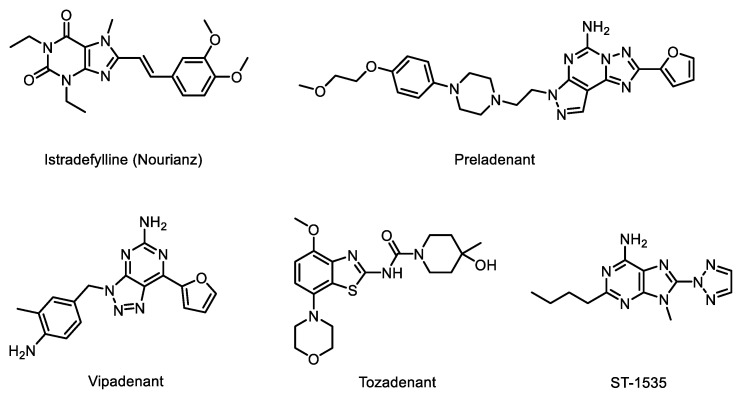
A_2A_ receptor antagonists as anti-Parkinson’s disease agents.

The chemical structures of five A_2A_ receptor antagonists that underwent clinical trials for PD are shown in Figure 1. Among them, istradefylline is a first-in-class adenosine A_2A_ receptor antagonist approved by the FDA and is currently in clinical use [3]. Preladenant developed by Merck was discontinued due to negative outcomes in a phase III trial, and vipadenant developed by Vernalis plc/Biogen Idec was halted due to toxicity. Moreover, the development of tozadenant from Acorda Therapeutics was halted at phase III due to agranulocytosis and associated serious adverse events [4]. ST-1535, which was developed by Sigma Tau, was also discontinued, probably due to the presence of an active metabolite.

Istradefylline is highly selective for the A_2A_ receptor and much less selective for the other adenosine receptors A_1_, A_2B_ and A_3_ [5]. In the brain, presynaptic A_1_ receptors on dopaminergic neurons may function synergistically with A_2A_ receptors in the presence of antagonists: inhibition of the A_1_ receptor will facilitate dopamine release, while inhibition of the A_2A_ receptor will enhance postsynaptic responses to dopamine [6]. Thus, a highly plausible hypothesis is that a dual antagonist of the A_2A_/A_1_ receptor may exert even more beneficial effects on PD than a selective A_2A_ antagonist [7,8].

**Figure 2:**
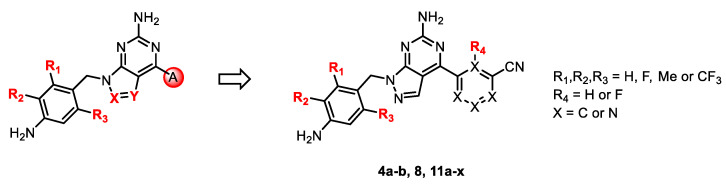
Design of the core structure.

In this study, based on the aforementioned hypothesis, we designed and synthesized novel dual A_2A_/A_1_ antagonists with a 1*H*-pyrazolo[3,4*-d*]pyrimidin-6-amine core scaffold (**11o**) that has high brain permeability and metabolic stability, along with outstanding safety and pharmacokinetic (PK) profiles. Preladenant and vipadenant exhibited better in vitro activities than istradefylline but were less metabolically stable with relatively shorter PK profile due to the exposed furan on the right wing. We introduced a phenyl or pyridyl ring in place of the furan ring to compensate for this shortcoming (Figure 2). The comparatively low activity observed after replacing the furan ring with the phenyl ring was overcome by introducing a nitrile group on the ring. Similar result was obtained after replacing this group with pyridyl ring, and similar activity compared to the phenyl ring was detected when nitrile group was introduced. Indeed, the activity was maintained only when the nitrile group was placed in the meta position.

For core structure design, we initially investigated the representative compounds with core bicyclic ring compounds in which triazole, pyrazole and imidazole were fused to pyrimidine (Figure 2). Among them, the pyrazolopyrimidine ring yielded the best physicochemical property and required the simplest synthetic route. Hence, in the present study, a series of derivatives with pyrazolopyrimidine core structures were investigated as A_2A_ and A_1_ receptor antagonists (**4a-b, 8, 11a-x**) and drug candidate **11o** was finally discovered as a potential treatment for Parkinson’s disease.

## 2. Results and Discussion

### 2.1. Chemistry

**Scheme 1:**
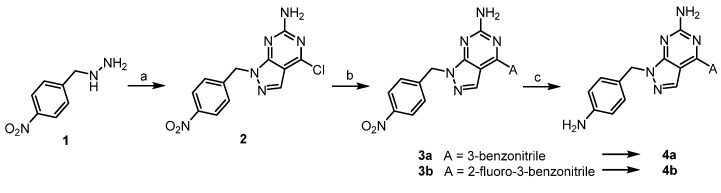
Synthesis of derivatives **4a-b**. **Reagents and conditions:** (**a**) 2-amino-4,6-dichloropyrimidine-5-carboxaldehyde, DMF, TEA, 0–25 °C, 1 h, crude; (**b**) boronic acid pinacol ester (A), Pd(PPh_3_)_4_, Na_2_CO_3_, dioxane/H_2_O, 100 °C, 3 h, crude; (**c**) Fe/NH_4_Cl, THF/H_2_O, 80 °C, 2 h, 49–51%.

**Scheme 2:**
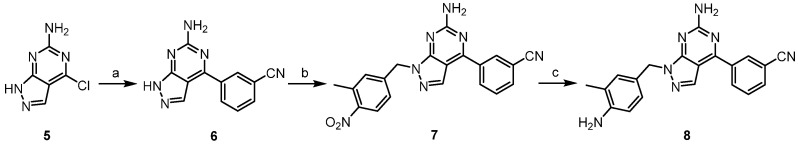
Synthesis of derivative **8**. **Reagents and conditions:** (**a**) 3-cyanophenylboronic acid, Pd(PPh_3_)_4_, Na_2_CO_3_, dioxane/H_2_O, 100 °C, 16 h, crude; (**b**) 3-methyl-4-nitrobenzyl chloride, K_2_CO_3_, DMF, 80 °C, 16 h, 24%; (**c**) Fe/NH_4_Cl, EtOH/H_2_O, 60 °C, 1 h, 38%.

**Scheme 3:**
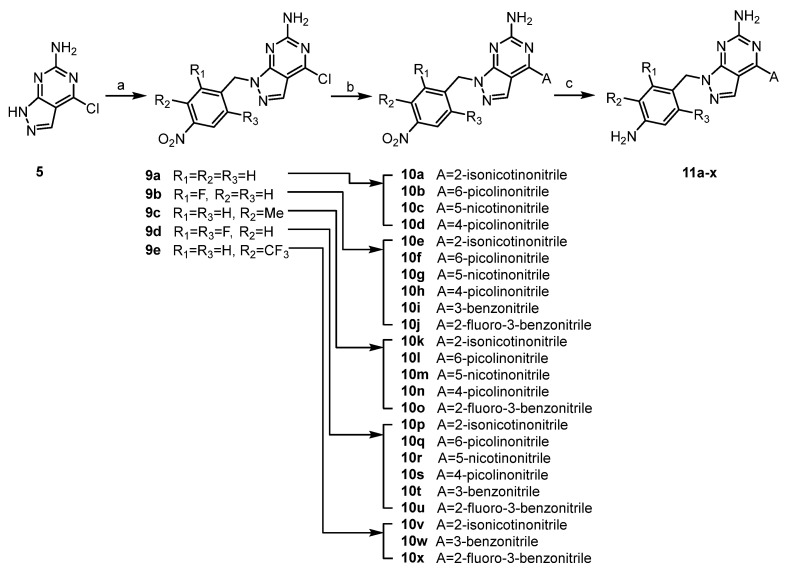
Synthesis of derivatives **11a-x**. **Reagents and conditions:** (**a**) ArCH_2_X, K_2_CO_3_, DMF, 80 °C, 16 h, 37–74%; (**b**) (method A) boronic acid (A), Pd(PPh_3_)_4_, Na_2_CO_3_, dioxane/H_2_O, 110 °C, 16 h, (method B) sodium cyanopyridine-sulfinate, Pd(OAc)_2_, K_2_CO_3_, PCy_3_, dioxane, 120 °C, 16 h, 21–90%; (**c**) Fe/NH_4_Cl, EtOH/H_2_O, 60 °C, 1h, 7–68%.

The synthesis of A_2A_ and A_1_ receptor antagonists with 1*H*-pyrazolo [3,4*-d*]pyrimidin-6-amine as a core scaffold (compounds **4a-b, 8, 11a-x**) was accomplished using three methods with a short three-step route. The general procedures for the three methods are described in Scheme 1, Scheme 2 and Scheme 3.

For the synthesis of compounds **4a** and **4b**, the core bicyclic ring was synthesized directly using hydrazine (Scheme 1). The core scaffold, 1*H*-pyrazolo[3,4*-d*]pyrimidin-6-amine, was generated through ring condensation by reacting hydrazine with 2-amino-4,6-dichloropyrimidine-5-carboxaldehyde. Since the pyrimidine reagent has two chloro atoms symmetrical on both sides, the resulting hydrazine obtained after condensing with aldehyde reacts with any chloro group on both sides of the pyrimidine to obtain the same intermediate **2**. The chlorine of compound **2** was substituted with 3-benzonitrile groups by Suzuki coupling, and then nitro reduction with iron dust provided the final compounds.

For the synthesis of compound **8**, commercially available 4-chloro-1*H*-pyrazolo[3,4*-d*]pyrimidin-6-amine (**5**) was reacted with 3-cyanophenylboronic acid by Suzuki coupling to provide compound **6**. Alkylation with benzyl halide followed by nitro reduction provided the final product (Scheme 2).

For the synthesis of compounds **11a**-**11x**, intermediate **5** was first alkylated with the corresponding benzyl halides, and then substituted phenyl/pyridyl was incorporated by Pd-catalyzed cross-coupling in a different order from Scheme 3. For ortho-substituted cyanopyridyls (A1 and A2, Table 1), classic Suzuki, Stille and Negishi coupling did not work well due to strong electron deficiency. This deficit was resolved by applying Pd-catalyzed desulfination coupling [9,10]. Finally, nitro reduction with iron dust provided the final products. Notably, during nitro reduction, the cyano group of pyridyl was also reduced in Fe/NH_4_Cl and then readily hydrolyzed. A similar observation was recorded under Pd/C hydrogenolysis conditions. However, the problem was solved by shortening the reduction time in Fe/NH_4_Cl, and the reaction was generally completed within 1 h. All final compounds were efficiently purified to >95% purity using normal phase prep-HPLC.

### 2.2. Biological Activities

#### 2.2.1. In Vitro Activity against Human A_1_ and A_2A_ Receptors

The binding affinities of synthesized compounds to human A_2A_ receptors (*h*A_2A_R) were measured by assessing competitive binding of ligands to radioligand [^3^H]-DPCPX using membrane preparations of human recombinant HEK-293 cells expressing human A_2A_ receptors [11]. As shown in Table 1, we investigated the structure–activity relationship (SAR) for the **A**-group (cyanopyridyl or cyanophenyl) and the R_1_, R_2_ and R_3_ of benzylamine substituted to the core scaffold toward human A_2A_ receptors (*h*A_2A_R).

In the SAR of R_1-3_ on the benzylamine, compounds with unsubstituted benzylamine rings (**11a**-**d** and **4a**-**b**) showed a low binding affinity to the *h*A_2A_ receptor, whereas CF_3_ on R_2_ showed the best result. The order of change in activity was CF_3_ > *di-*F > Me > F > H. A similar pattern was obtained for both pyridyl and phenyl groups, irrespective of the **A** group.

Next, we examined the SAR of **A** group. In the pyridyl **A** group, the activity was substantially affected by the position of the ‘N’ on the pyridyl ring. Among the series of compounds **A1–6**, 6-picolinonitrile derivatives (**A2**) showed the best activity, followed by 2-isonicotinonitrile-substituted compounds (**A1**) with moderate activity. 5-Nicotinotrile (**A3**) and 4-picolinonitrile derivatives (**A4**) displayed very low activity. In the phenyl **A**-region, they all exhibited promising activity. Little significant change in activity was observed between 3-benzonitrile (**A5**) and 2-fluoro-3-benzonitrile (**A6**). Overall, the cyanophenyl group showed slightly better activity than the cyanopyridyl group in the SAR of the **A** group.

Among the compounds tested, all compounds with **A2**, **A5** and **A6** groups displayed good activities. However, cyanopyridyl groups **A1**–**A4** showed less inhibitory activity toward *h*A_1_ receptor than compounds with cyanophenyl groups (**A5** and **A6**, unpublished data). Recent findings reported using PD animal models suggested that affinity for *h*A_1_ receptors might positively contribute to PD [6,8]. Therefore, we hypothesized that the use of cyanophenyl groups (**A5** and **A6**) may exert a higher synergistic effect than cyanopyridyl (**A2**) on PD.

From the list of compounds with K_i_ value of less than 30 nM for A_2A_ receptor, we have further selected for compounds with K_i_ value below 100 nM for A_1_ receptor. In addition, it was deemed necessary to select a highly soluble compound because only intranasal delivery was considered to maximize compound’s brain exposure. Compounds with CF_3_, a strong electron withdrawing group (EWG), next to aniline, such as **11v**, **11w** and **11x**, have greatly reduced solubility due to increased difficulties in forming salt. In the case of **11t** and **11u** where fluoro group was substituted in R_1_ and R_2_, salt formation was also not feasible due to strong EWG. Among the selected compounds capable of salt formation, **11o** displayed the best binding affinity for both A_2A_ and A_1_ receptors. Thus, we have selected **11o** as a potential drug candidate and conducted further studies.

**Figure 3:**
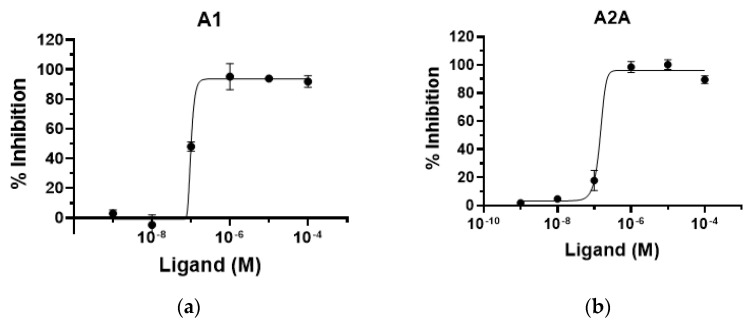
Functional activity of compound **11o** toward human adenosine receptors. (**a**) A_1_ receptor in CHO–K1 cell. (**b**) A_2A_ receptor in HEK–293 cell.

Next, the functional activity of **11o** against A_1_ and A_2A_ adenosine receptors was evaluated using c-AMP assay in CHO-K1 (A_1_) and HEK-293 (A_2A_) cells. As shown in Figure 3, **11o** displayed a full antagonism with IC_50_ values of 98.8 nM and 136 nM in A_1_ and A_2A_ adenosine receptors, respectively, indicating that **11o** is a dual A_1_/A_2A_ antagonist.

#### 2.2.2. Selectivity for Adenosine Receptor Subtypes

We examined and compared the binding activities of **11o** toward four adenosine receptors, A_1_, A_2A_, A_2B_ and A_3_, to investigate its selectivity for adenosine receptors.

Whereas **11o** showed relatively high selectivity toward both receptors A_1_ and A_2A_, with K_i_ values of 55 nM and 13.3 nM, respectively, it exerted low binding affinities on A_2B_ and A_3_ receptors with K_i_ values 0.4 μM and 1.05 μM, respectively, indicating that compound **11o** is a dual specific A_2A_/A_1_ receptor antagonist (Table 2).

#### 2.2.3. In Vivo Activity in Animal Models of Parkinson’s Disease

In a subsequent study, we investigated the therapeutic efficacy of **11o** in PD using an MPTP-lesioned mouse model [12]. This MPTP-lesion mouse model is one of the most well-established PD models and causes an approximately 90% loss of striatal dopamine, as analyzed using HPLC 7 days after the administration of the last dose of MPTP [13].

**Figure 4:**
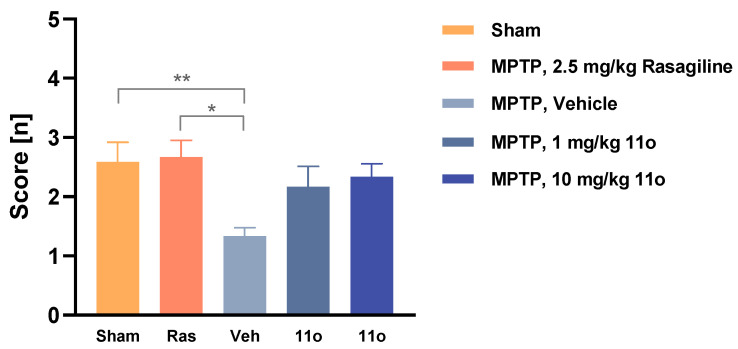
Nest-building score of sham or MPTP-lesioned mice treated either with vehicle, rasagiline or compound **11o** (intranasal). The graph presents the mean nesting scores ± SEM (*n* = 12 rats per group). Data were analyzed using the Kruskal–Wallis test and Dunn’s multiple comparison test (all mean values were compared to MPTP, vehicle). * *p* < 0.05 and ** *p* < 0.01.

When **11o** was administered 30 min after the last MPTP treatment and once daily for 7 days thereafter, it was unable to rescue dopamine depletion caused by MPTP (data not shown) at doses up to 10 mg/kg. However, when mice were tested for nest-building activity after the intranasal administration of 1 and 10 mg/kg doses, compound **11o** partially restored the activity level to near sham level, although a significant difference was not observed (Figure 4). Taken together, compound **11o** positively affected the behavioral outcome of MPTP-lesioned mice without affecting the dopamine level. An interesting approach would be to determine the synergistic effect of L-DOPA and **11o** in a 6-OHDA rat model, which is currently being investigated by our group.

**Figure 5:**
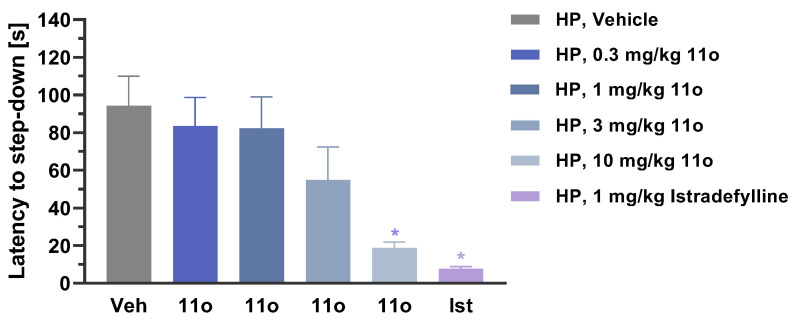
Latency to step-down in female rats injected with haloperidol (HP). Rats were administered with **11o** (PO) 60 min before haloperidol injection at increasing dose levels. Istradefylline (1 mg/kg, PO) was used as positive control. The graph presents the mean latency (seconds) ± SEM (*n* = 10 rats per group). Data were analyzed using the one-way ANOVA with Dunnett’s multiple comparisons test (all mean values were compared to vehicle). * *p* < 0.01.

In another experiment, compound **11o** resulted in a dose-dependent reversal of catalepsy in female rats induced by haloperidol (Figure 5). Vehicle or varying amount of **11o** (0.3, 1, 3, 10 mg/kg) was administered first, followed by IP injection of 1 mg/kg haloperidol 60 min later. When catalepsy was assessed 60 min after haloperidol injection by measuring latency to step down from the rod, **11o** dose-dependently reversed haloperidol-induced catalepsy, with maximum effect observed at 10 mg/kg, which was comparable to the effect observed with positive control istradefylline (1 mg/kg).

### 2.3. Pharmacokinetic Studies

#### 2.3.1. In Vitro Metabolic Stability

In vitro intrinsic clearance (CL_int_) was investigated using cryopreserved hepatocytes from five different species—CD-1 mice, Sprague Dawley rats, beagle dogs, cynomolgus monkeys and humans—to understand the metabolic stability of **11o** (Table 3).

When 1 μM **11o** was incubated with hepatocytes from five species, it disappeared with t_1/2_ values of 70.9, 46.9, 124 and 184 min after incubation in mouse, rat, monkey and human hepatocytes, respectively. Under this test condition, the compound was stable in dog hepatocytes where half-life, intrinsic clearance and extrapolated clearance values were undetermined. Using the well-established model of hepatic elimination, these half-lives resulted in CL_int_ values of 9 mL/min/million cells of 0.0098 (mouse), 0.0148 (rat), 0.056 (monkey), and 0.0038 (human). The extrapolated CL_int_ values (mL/min/kg) [14] were 115 (mouse), 69.2 (rat), 20.1 (monkey) and 9.6 (human). The CL_int_ of compound **11o** in human hepatocytes was less than 40% of midazolam, which was used as a positive control in this experiment. Based on the half-life, the stability of **11o** in hepatocytes followed the rank order of dog >> human > monkey > mouse > rat, indicating that **11o** was very stable in dog hepatocytes, while it was least stable in rats.

#### 2.3.2. In Vivo Pharmacokinetic Study

**Figure 6:**
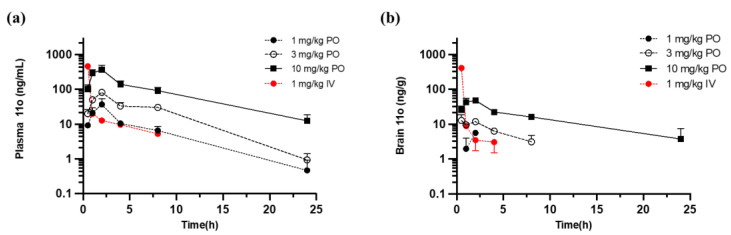
Plots showing the mean concentrations of **11o** versus time in mouse plasma (**a**) and brain (**b**). **11o** was administered either orally (PO) or intravenously (IV). Three mice were sampled from each group for blood and brain collection at each time point. Plasma and brain samples were analyzed for **11o** concentrations using LC–MS/MS.

When **11o** was administered orally to mice, it showed a dose-dependent increase in plasma concentration (Figure 6a). In addition, both oral and intravenous administration resulted in detectable and dose proportional levels of **11o** in the brain (Figure 6b), indicating good brain penetration.

The pharmacokinetic parameters of **11o** in mouse plasma are described in Table 4. T_max_ was 2 h for all doses following PO dosing. T_1/2_ values were 4.38 h, 3.66 h and 5.69 h for doses of 1, 3 and 10 mg/kg, respectively. C_max_ values were 36.8 ng/mL, 81.4 ng/mL and 367 ng/mL for doses 1, 3 and 10 mg/kg, respectively, and AUC_last_ values were 176 h·ng/mL, 577 h·ng/mL and 2248 h·ng/mL for the three doses. Following IV dosing, T_max_ was 0.083 h, and the T_1/2_ value was 3.95 h. C_max_ and AUC_last_ values were 459 ng/mL and 332 h·ng/mL, respectively.

Intranasal (IN) dosing resulted in a shorter T_max_ of 0.5 h for all groups, indicating that the compound reached the system faster via this route compared to PO. The T_1/2_ value was 7.4 h for 10 mg/kg dose group. The T_1/2_ values for the other 2 dose groups were not calculated due to insufficient data points in the elimination phase or poor goodness-of-fit (R^2^ < 0.8) for the elimination phase. The C_max_ values were 23.3 ng/mL, 53.9 ng/m and 88.5 ng/mL for doses of 1, 3 and 10 mg/kg, respectively, with AUC_last_ values of 74.9, 183 and 444 h·ng/mL, respectively.

Oral bioavailability was assessed relative to the AUC_last_ for the IV dose group, and the data are presented in Table 5. Bioavailability of **11o** after oral dosing was 53%, 57.9% and 67.7% for the 1, 3 and 10 mg/kg dose groups, respectively, indicating good bioavailability. Bioavailability after IN administration was relatively poorer, ranging from 13.4–22.6%, depending on the dose.

The pharmacokinetic parameters of **11o** in the mouse brain are described in Table 5. Both PO and IN dosing resulted in detectable levels of **11o** in the brain, with significant AUC_last_ values of 4.26/5.23, 56.4/14.2 and 375/81.3 h·ng/mL for doses of 1, 3 and 10 mg/kg, respectively. The B/P ratio ranged from 0.0242–0.167 for PO dosing groups and 0.0698–0.183 for IN dosing groups. Based on these results, **11o** was estimated to reach brain faster, despite lower AUC_last_ when delivered through the IN route, when compared to the PO route.

### 2.4. Toxicity Studies

*h*ERG (human-ether-a-go-go) liability was investigated in an *h*ERG-expressing HEK cell line using the manual patch clamp method to assess the potential cardiotoxicity of **11o** [15]. The IC_50_ was 116 μM, suggesting a low *h*ERG liability of the compound at physiologically relevant dose (Table 6).

The mutagenic potential of **11o** was also tested by assessing its ability to reverse mutations in a bacterial mutagenesis study [16,17]. **11o** did not cause any positive mutagenic response when applied at up to 1867 μg/plate, suggesting that the compound is not mutagenic.

Next, we investigated the maximum tolerated dose (MTD) of **11o** in rats and dogs. In male and female Sprague Dawley rats, MTD following single oral administrations of **11o** was >1000 mg/kg, with no body weight loss or mortality observed. When MTD was assessed in male and female beagle dogs, the value obtained following single oral administrations of the compound was >400 mg/kg, with slight decreases in body weight in both males (−5.7%) and females (−6.5%) that were not considered adverse and test article-related. These results indicate that a single dose of **11o** was well-tolerated up to 1000 mg/kg and 400 mg/kg in rats and dogs, respectively.

## 3. Materials and Methods

### 3.1. Chemistry

For ^1^H and ^13^C NMR spectra Bruker Avance III 400 MHz spectrometer was used. The coupling constant was displayed as hertz and the peak multiplicities as s (singlet), d (doublet), dd (doublet of doublet), t (triplet), m (multiplet), br d (broad doublet), br s (broad singlet) and td (triplet of doublet) in the NMR. HPLC used to analyze the purity of chemicals was Agilent 1100 LC/G1956A (column: Agilent Eclipse Plus C18 3.5 μm, 4.6 × 150 mm); Agilent 1100 LC/G1956A (column: Waters Xbridge^®^C18 3.5 μm, 4.6 × 150 mm); Agilent 1200/G6410B (column: Eclipse Plus C18 3.5 μm 4.6 × 150 mm); SHIMADZU LC 20AB (column: Xbridge^®^C18 3.5 μm 4.6 × 150 mm). In some cases, prep-HPLC (column: Phenomenex Luna C18 150 × 25 mm × 10 μm; mobile phase: [water (0.225% FA)-CAN]; B%: 23–53%, 10 min) was used to purify chemicals. LC/MS was measured using SHIMADZU LCMS-2020 (column: kinetex EVO C18 2.1 × 30 mm, 5 μm), Agilent 1200/G6110A (column: ACE Excel 5 C18 2.1 × 30 mm, 5 μm). High-resolution mass spectra (HRMS) were measured by electrospray ionization (ESI) with an Agilent G6520 Q-TOF.

#### 3.1.1. General Procedure A for **4a-4b**

##### 4-Chloro-1-(4-nitrobenzyl)-1*H*-pyrazolo[3,4-*d*]pyrimidin-6-amine (**2**)

To a solution of 2-amino-4,6-dichloropyrimidine-5-carboxaldehyde (1.0 eq) in DMF TEA (5.0 eq) and 4-nitrophenylhydrazine **1** (1.0 eq, 2 HCl) were added at 0 °C, and then the mixture was stirred at 25 °C for 1 h. The reaction mixture was diluted with Ethyl acetate and washed with brine. The organic layer was concentrated to give a brown solid. Compound **2** (crude) was obtained as a yellow solid.; ^1^H NMR (400 MHz, DMSO-d_6_) δ 8.19 (d, *J* = 8.4 Hz, 2H), 8.09 (s, 1H), 7.40 (br d, *J* = 8.8 Hz, 4H), 5.56 (s, 2H).

##### 3-(6-Amino-1-(4-nitrobenzyl)-1*H*-pyrazolo[3,4-*d*]pyrimidin-4-yl)benzonitrile (**3a**)

A mixture of intermediate **2** (1.00 eq), 3-cyanophenylboronic acid pinacol ester (1.5 eq) Na_2_CO_3_ (2.0 eq) and Pd(PPh_3_)_4_ (0.05 eq) in H_2_O and dioxane was degassed and purged with N_2_ three times and the mixture was stirred at 100 °C for 3 h under N_2_ atmosphere. Reaction mixture was filtered to remove the insoluble. Filtrate was concentrated in vacuo to give residue. The residue was triturated by water. Then, the mixture was triturated by EtOH. Compound **3a** (crude) was obtained as a yellow solid.; ^1^H NMR (400 MHz, DMSO-d_6_) δ 8.59–8.44 (m, 3H), 8.20 (br d, *J* = 8.8 Hz, 2H), 8.07 (br d, *J* = 7.6 Hz, 1H), 7.80 (t, *J* = 7.6 Hz, 1H), 7.43 (br d, *J* = 8.8 Hz, 2H), 7.15 (br s, 2H), 5.62 (s, 2H).

##### 3-(6-Amino-1-(4-aminobenzyl)-1*H*-pyrazolo[3,4-*d*]pyrimidin-4-yl)benzonitrile (**4a**)

A mixture of intermediate 3 (1.0 eq), Fe (5.0 eq) and NH_4_Cl (5.0 eq) in THF and H_2_O was stirred at 80 °C for 2 h. The mixture was filtrated and concentrated to give residue. The cake was quenched with 1 N HCl at 25 °C. The residue was purified by column chromatography (SiO_2_, dichloromethane/tetrahydrofuran = 1/0 to 10/1). Product 4a (51.3% yield, 99.2% purity) was obtained as a yellow solid.; ^1^H NMR (400 MHz, DMSO-d_6_) δ 8.52–8.46 (m, 2 H), 8.36 (s, 1 H), 8.06–8.04 (m, 1 H), 7.79 (t, *J* = 7.8 Hz, 1 H), 7.08 (s, 2 H), 6.97 (d, *J* = 8.4 Hz, 2 H), 6.50 (dd, *J* = 6.8, 1.6 Hz, 2 H), 5.25 (s, 2 H), 5.05 (s, 2 H); ^13^C NMR (100 MHz, DMSO-d_6_) δ = 162.5, 158.6, 156.4, 148.6, 138.2, 134.8, 133.6, 133.5, 132.2, 130.7, 129.1, 124.6, 118.9, 114.1, 112.6, 105.0, 49.7; HRMS (ESI) calc. for C_19_H_15_N_7_ [M + H]^+^ 341.1389, found: 341.1386.

##### 3-(6-Amino-1-(4-aminobenzyl)-1*H*-pyrazolo[3,4-*d*]pyrimidin-4-yl)-2-fluorobenzonitrile (**4b**)

Compounds **3b** and **4b** were sequentially processed according to general procedure A to yield **4b** as yellow solid (91.0 g, 252 mmol, 49.0% yield, 98.27% purity).; ^1^H NMR (400 MHz, DMSO-d_6_) δ 8.18–8.12 (m, 2 H), 7.98 (d, *J* = 3.6 Hz, 1 H), 7.60 (t, *J* = 7.8 Hz, 1 H), 7.13 (s, 2 H), 6.98 (d, *J* = 8.4 Hz, 2 H), 6.50 (d, *J* = 8.4 Hz, 2 H), 5.24 (s, 2 H), 5.06 (s, 2 H); ^13^C NMR (100 MHz, DMSO-d_6_) δ 162.6, 161.8, 159.2, 155.6 (d, *J* = 15.9 Hz, 1C), 148.7, 137.0 (d, *J* = 3.5 Hz, 1C), 136.3, 133.8 (d, *J* = 8.3 Hz, 1C), 129.1, 126.3 (d, *J* = 3.9 Hz, 1C), 126.1 (d, *J* = 11.9 Hz, 1C), 124.5, 114.4, 114.1, 106.8, 102.0 (d, *J* = 15.9 Hz, 1C), 49.7; HRMS (ESI) calc. for C_19_H_14_FN_7_ [M + H]^+^ 359.1295, found: 359.1289.

#### 3.1.2. General Procedure B for **8**

##### 3-(6-Amino-1*H*-pyrazolo[3,4-*d*]pyrimidin-4-yl)benzonitrile (**6**)

A mixture of commercially available 4-chloro-1H-pyrazolo[3,4-d]pyrimidin-6-amine 5 (1.0 eq), (3-cyanophenyl)boronic acid (1.2 eq), Pd(PPh_3_)_4_ (0.1 eq) and Na_2_CO_3_ (2 eq) in dioxane was degassed and purged with nitrogen for three times, and then the mixture was stirred at 100 °C for 16 h under nitrogen atmosphere. Reaction mixture was partitioned between ethyl acetate and water. The organic phase was separated, washed by brine, dried over sodium sulfate, filtered and concentrated under reduced pressure to give residue. Compound 6 (500 mg, crude) was obtained as a yellow solid and used in the next step without further purification.: MS: m/z = 237.1 (M + 1, ESI^+^).

##### 3-[6-Amino-1-[(3-methyl-4-nitro-phenyl)methyl]pyrazolo[3,4-*d*]pyrimidin-4-yl]benzonitrile (**7**)

To a solution of 4-(chloromethyl)-2-methyl-1-nitro-benzene (500 mg, 1.27 eq) and 3-(6-amino-1*H*-pyrazolo[3,4-*d*]pyrimidin-4-yl)benzonitrile (1.0 eq) in DMF, K_2_CO_3_ (2.0 eq) was added. The mixture was stirred at 80 °C for 16 h. The reaction mixture was concentrated under reduced pressure to give residue. The residue was purified by prep-HPLC (column: Phenomenex Luna C18 150 × 25mm × 10μm; mobile phase: [water (0.225% FA)-ACN]; B%: 40–70%, 10 min). Compound **7** (200 mg, 24% yield) was obtained as a yellow solid.; ^1^H NMR (400 MHz, DMSO-d_6_) δ 8.56–8.42 (m, 3H), 8.10–8.06 (m, 1H), 8.00–7.92 (m, 1H), 7.86–7.76 (m, 1H), 7.40–7.30 (m, 1H), 7.24–7.08 (m, 3H), 5.65–5.43 (m, 2H), 2.49 (s, 3H); MS: m/z = 386.0 (M + 1, ESI^+^).

##### 3-(6-Amino-1-(4-amino-3-methylbenzyl)-1*H*-pyrazolo[3,4-*d*]pyrimidin-4-yl)benzonitrile (**8**)

To a solution of intermediate **7** (150 mg, 1.0 eq) in ethanol (12 mL) and water (4 mL), iron dust (5.0 eq) and NH_4_Cl (8.0 eq) were added. The mixture was stirred at 60 °C for 1 h. The reaction mixture was filtered and concentrated under reduced pressure to give the residue. The resulting residue was purified by prep-HPLC (column: Phenomenex Luna C18 150 × 25 mm*10 μm; mobile phase: [water (0.225% FA)-ACN]; B%: 23–53%, 10 min). The product **8** (54.26 mg, 38% yield, 98.27% purity) was obtained as a white solid.; ^1^H NMR (400 MHz, DMSO-d_6_) δ 8.51 (s, 1H), 8.47 (d, *J* = 8.0 Hz, 1H), 8.35 (s, 1H), 8.06 (d, *J* = 7.6 Hz, 1H), 7.79 (t, *J* = 7.8 Hz, 1H), 7.05 (s, 2H), 6.88 (s, 1H), 6.83 (d, *J* = 8.4 Hz,1H), 6.52 (d, *J* = 8.0 Hz,1H), 5.23 (s, 2H), 4.80–4.78 (m, 2H), 1.99 (s, 3H); ^13^C NMR (100 MHz, DMSO-d_6_) δ = 162.0, 158.1, 155.9, 146.1, 137.8, 134.4, 133.1, 133.0, 131.8, 130.3, 129.6, 126.2, 124.4, 120.9, 118.4, 113.7, 112.1, 104.5, 49.2, 17.5; HRMS (ESI) calc. for C_20_H_17_N_7_ [M + H]^+^ 355.1545, found: 355.1544.

#### 3.1.3. General Procedure C for **11a~11w**

##### 4-Chloro-1-(3-methyl-4-nitrobenzyl)-1H-pyrazolo[3,4-d]pyrimidin-6-amine (**9c**)

To a solution of 4-chloro-1*H*-pyrazolo[3,4-*d*]pyrimidin-6-amine **5** (1.0 eq) and 4-(chloromethyl)-2-methyl-1-nitro-benzene (0.8 eq) in DMF was added K_2_CO_3_ (2.0 eq) and stirred at 80 °C for 16 h. The reaction mixture was allowed to room temperature, filtered and concentrated under reduced pressure to give residue. The crude compound **9c** was obtained as a yellow solid (800 mg, crude) and used in the next step without further purification; ^1^H NMR (400 MHz, DMSO-d_6_) δ 8.08 (s, 1H), 7.96 (d, *J* = 8.4 Hz, 1H), 7.40 (s, 2H), 7.32 (s, 1H), 7.20 (dd, *J* = 8.6, 1.4 Hz, 1H), 5.50 (s, 2H), 2.49 (s, 3H); ^13^C NMR (100 MHz, DMSO-d_6_) δ 161.65, 156.06, 153.70, 148.06, 142.45, 133.17, 132.96, 131.27, 125.87, 124.87, 106.19, 48.92, 19.57; HRMS (ESI) calc. for C_13_H_12_ClN_6_O_2_ [M + H]^+^ 319.0705, found: 319.0708.

##### 3-(6-Amino-1-(4-amino-3-methylbenzyl)-1*H*-pyrazolo[3,4-*d*]pyrimidin-4-yl)-2-fluorobenzonitrile (**10o**)

A mixture of (3-cyano-2-fluoro-phenyl)boronic acid (1.2 eq), intermediate **9c** (1.0 eq), Pd(PPh_3_)_4_ (0.1 eq) and Na_2_CO_3_ (2.0 eq) in dioxane and water was degassed and purged with nitrogen three times and was stirred at 110 °C for 16 h under nitrogen atmosphere. The reaction mixture was filtered and concentrated under reduced pressure to give residue. The resulting residue was purified by flash silica-gel chromatography (ISCO^®^; 40 g SepaFlash^®^ Silica Flash Column, eluent of 0–80% EtOAc/petroleum ether gradient @ 40 mL/min). Compound **10o** was obtained as a yellow solid (90% yield, 98.54 % purity). ^1^H NMR (400 MHz, DMSO-d_6_) δ 8.22–8.18 (m, 2H), 8.11 (d, *J* = 4.4 Hz, 1H), 8.00 (d, *J* = 11.2 Hz, 1H), 7.64 (t, *J* = 10.2 Hz, 1H), 7.39 (s, 1H), 7.24–7.22 (m, 3H), 5.56 (s, 2H), 2.52 (s, 3H); ^13^C NMR (100 MHz, DMSO-d_6_) δ 162.32, 161.35, 158.74, 155.85, 155.32, 148.02, 142.77, 136.53 (d, *J* = 3.6 Hz, 1C), 135.88, 134.22 (d, *J* = 8.0 Hz, 1C), 133.18, 131.39, 125.95–125.83 (m, 1C), 125.53 (d, *J* = 12.5 Hz, 1C), 124.90, 113.82, 106.23, 101.58 (d, *J* = 15.0 Hz, 1C), 48.56, 19.64; HRMS (ESI) calc. for C_20_H_15_FN_7_O_2_ [M + H]^+^ 404.1266, found: 404.1268.

##### 3-(6-Amino-1-(4-amino-3-methylbenzyl)-1*H*-pyrazolo[3,4-*d*]pyrimidin-4-yl)-2-fluorobenzonitrile (**11o**)

To a solution of intermediate **10o** (1.0 eq) in water and ethanol, iron dust (5.0 eq) and NH_4_Cl (8.0 eq) were added. Mixture was stirred at 60 °C for 1 h. Reaction mixture was then filtered and concentrated under reduced pressure to give residue. The resulting residue was purified by prep-HPLC (column: Phenomenex Synergi C18 150 × 25 mm × 10 μm; mobile phase: [water (0.225%FA)-ACN]; B%: 16–46%, 11 min), after which 89% purity compound was obtained. The residue was further purified by prep-HPLC (column: Phenomenex Luna C18 150 × 25 mm × 10 μm; mobile phase: [water (0.225% FA)-ACN]; B%: 18–48%,10 min). Compound **11o** was obtained as a white solid (59% yield, 97.78% purity); ^1^H NMR (400 MHz, DMSO-d_6_) δ 8.17–8.13 (m, 2H), 7.96 (d, *J* = 3.6 Hz, 1H), 7.60 (t, *J* = 7.6 Hz, 1H), 7.11 (s, 2H), 6.89 (d, *J* = 1.6 Hz, 1H), 6.84 (dd, *J* = 8.0, 2.0 Hz, 1H), 6.52 (d, *J* = 8.0 Hz, 1H), 5.22 (d, *J* = 4.8 Hz, 2H), 4.81 (br s, 2H), 2.00 (s, 3H); ^13^C NMR (100 MHz, DMSO-d_6_) δ 162.2, 161.3, 158.7, 155.1 (d, *J* = 15.8 Hz, 1C), 146.1, 136.6, 135.8, 133.3 (d, *J* = 8.4 Hz, 1C), 129.7, 126.2, 125.9, 125.7 (d, *J* = 12.5 Hz, 1C), 124.3, 120.9, 113.9, 113.7, 106.3, 101.5 (d, *J* = 15.9 Hz, 1C), 49.2, 17.4; HRMS (ESI) calc. for C_20_H_16_FN_7_ [M + H]^+^ 373.1451, found: 373.1466.

##### 2-(6-Amino-1-(4-aminobenzyl)-1*H*-pyrazolo[3,4-*d*]pyrimidin-4-yl)isonicotinonitrile (**11a**)

Compounds **9a** and **10a** were sequentially processed according to general procedure C to yield **11a** as yellow solid (8% yield, 95.02% purity); ^1^H NMR (400 MHz, DMSO-d_6_) δ 9.08 (dd, *J* = 5.2, 0.8 Hz, 1H), 8.67 (t, *J* = 1.4 Hz, 1H), 8.45 (s, 1H), 8.07 (dd, *J* = 4.8, 1.6 Hz, 1H), 7.10 (s, 2H), 6.95 (d, *J* = 8.8 Hz, 2H), 6.48 (dd, *J* = 6.8, 1.6 Hz, 2H), 5.24 (s, 2H), 5.03 (s, 2H); ^13^C NMR (100 MHz, DMSO-d_6_) δ = 162.0, 156.6, 156.4, 155.7, 151.1, 148.2, 134.8, 128.5, 127.1, 124.1, 123.6, 120.6, 116.6, 113.7, 104.4, 49.1; HRMS (ESI) calc. for C_18_H_14_N_8_ [M + H]^+^ 342.1341, found: 342.1334.

##### 6-(6-Amino-1-(4-aminobenzyl)-1*H*-pyrazolo[3,4-*d*]pyrimidin-4-yl)picolinonitrile (**11b**)

Compounds **9a** and **10b** were sequentially processed according to general procedure C to yield **11b** as yellow solid (11% yield, 98.25% purity); ^1^H NMR (400 MHz, DMSO-d_6_) δ 8.65 (dd, *J* = 8.0, 1.2 Hz, 1H), 8.39 (s, 1H), 8.31 (t, *J* = 8.0 Hz, 1H), 8.24 (dd, *J* = 7.6, 1.2 Hz, 1H), 7.12 (s, 2H), 6.94 (d, *J* = 8.8 Hz, 2H), 6.48 (d, *J* = 8.4 Hz, 2H), 5.26 (s, 2H), 5.03 (s, 2H); ^13^C NMR (100 MHz, DMSO-d_6_) δ 162.0, 155.8, 148.1, 139.5, 134.5, 132.6, 130.7, 128.8, 129.2, 125.5, 124.1, 113.7, 104.4, 49.1; HRMS (ESI) calc. for C_18_H_14_N_8_ [M + H]^+^ 342.1341, found: 342.1334.

##### 5-(6-Amino-1-(4-aminobenzyl)-1*H*-pyrazolo[3,4-*d*]pyrimidin-4-yl)nicotinonitrile (**11c**)

Compounds **9a** and **10c** were sequentially processed according to general procedure C to yield **11c** as yellow solid (7% yield, 98.17% purity); ^1^H NMR (400 MHz, DMSO-d_6_) δ 9.53 (d, *J* = 2.0 Hz, 1H), 9.22 (d, *J* = 2.0 Hz, 1H), 8.91 (t, *J* = 2.0 Hz, 1H), 8.44 (s, 1H), 7.12 (s, 2H), 6.96 (d, *J* = 8.8 Hz, 2H), 6.48 (d, *J* = 8.4 Hz, 2H), 5.24 (s, 2H), 5.03 (s, 2H); ^13^C NMR (100 MHz, DMSO-d_6_) δ 162.0, 156.0, 155.8, 154.0, 152.4, 148.2, 139.4, 133.1, 132.3, 128.6, 124.0, 116.6, 113.6, 109.6, 104.8, 49.2; HRMS (ESI) calc. for C_18_H_14_N_8_ [M + H]^+^ 342.1341, found: 342.1345.

##### 4-(6-Amino-1-(4-aminobenzyl)-1*H*-pyrazolo[3,4-*d*]pyrimidin-4-yl)picolinonitrile (**11d**)

Compounds **9a** and **10d** were sequentially processed according to general procedure C to yield **11d** as white solid (18% yield, 95.28% purity); ^1^H NMR (400 MHz, DMSO-d_6_) δ 8.97 (d, *J* = 5.2 Hz, 1H), 8.60 (d, *J* = 0.8 Hz, 1H), 8.44 (s, 1H), 8.40 (dd, *J* = 5.0, 1.8 Hz, 1H), 7.19 (s, 2H), 6.95 (d, *J* = 8.4 Hz, 2H), 6.48 (d, *J* = 8.4 Hz, 2H), 5.25 (s, 2H), 5.04 (s, 2H); ^13^C NMR (100 MHz, DMSO-d_6_) δ 162.0, 156.0 (d, *J* = 2.5 Hz, 1C), 152.2, 148.2, 145.2, 133.6, 132.8, 128.6, 127.4, 126.4, 123.9, 117.4, 113.6, 104.8, 49.2; HRMS (ESI) calc. for C_18_H_14_N_8_ [M + H]^+^ 342.1341, found: 342.1365.

##### 2-(6-Amino-1-(4-amino-2-fluorobenzyl)-1*H*-pyrazolo[3,4-*d*]pyrimidin-4-yl)isonicotinonitrile (**11e**)

Compounds **9b** and 1**0e** were sequentially processed according to general procedure C to yield **11e** as yellow solid (32% yield, 96.12% purity); ^1^H NMR (400 MHz, DMSO-d_6_) δ 9.08 (dd, *J* = 4.8, 0.8 Hz, 1H), 8.68 (d, *J* = 1.2 Hz, 1H), 8.46 (s, 1H), 8.08 (dd, *J* = 5.2, 1.6 Hz, 1H), 7.13 (s, 2H), 6.88 (t, *J* = 8.4 Hz, 1H), 6.32–6.31 (m, 1H), 6.29 (s, 1H), 5.40 (s, 2H), 5.29 (s, 2H); ^13^C NMR (100 MHz, DMSO-d_6_) δ 162.6, 162.4, 160.2, 157.0, 156.1, 151.6, 151.0 (d, *J* = 11.6 Hz, 1C), 135.5, 131.2 (d, *J* = 6.7 Hz, 1C), 127.6, 124.1, 121.1, 117.1, 110.4, 110.2 (d, *J* = 2.5 Hz, 1C), 104.8, 100.2 (d, *J* = 25.05 Hz, 1C), 43.2; HRMS (ESI) calc. for C_18_H_13_FN_8_ [M + H]^+^ 360.1247, found: 360.1253.

##### 6-[6-Amino-1-[(4-amino-2-fluoro-phenyl)methyl]pyrazolo[3,4-*d*]pyrimidin-4-yl]pyridine-2-carbonitrile (**11f**)

Compounds **9b** and **10f** were sequentially processed according to general procedure C to yield **11f** as yellow solid (30% yield, 96.38% purity); ^1^H NMR (400 MHz, DMSO-d_6_) δ 8.65 (dd, *J* = 8.0, 1.2 Hz, 1H), 8.39 (s, 1H), 8.31 (t, *J* = 7.8 Hz, 1H), 8.24 (dd, *J* = 8.0, 1.2 Hz, 1H), 7.13 (s, 2H), 6.86 (t, *J* = 8.8 Hz, 1H), 6.31 (dd, *J* = 5.4, 1.8 Hz, 1H), 6.28 (s, 1H), 5.40 (s, 2H), 5.29 (s, 2H); ^13^C NMR (100 MHz, DMSO-d_6_) δ 162.1, 162.0, 159.7, 156.6, 156.5, 155.8, 150.5 (d, *J* = 11.5 Hz, 1C), 139.5, 134.7, 132.6, 130.6 (d, *J* = 6.6 Hz, 1C), 125.5, 117.3, 109.9, 109.8 (d, *J* = 2.4 Hz, 1C), 104.3, 99.8 (d, *J* = 23.8 Hz, 1C), 42.8 (d, *J* = 3.2 Hz, 1C); HRMS (ESI) calc. for C_18_H_13_FN_8_ [M + H]^+^ 360.1247, found: 360.1238.

##### 5-(6-Amino-1-(4-amino-2-fluorobenzyl)-1*H*-pyrazolo[3,4-*d*]pyrimidin-4-yl)nicotinonitrile (**11g**)

Compounds **9b** and **10g** were sequentially processed according to general procedure C to yield **11g** as yellow solid (13% yield, 94.57% purity); ^1^H NMR (400 MHz, DMSO-d_6_) δ 9.54 (d, *J* = 2.4 Hz, 1H), 9.23 (d, *J* = 2.0 Hz, 1H), 8.91 (t, *J* = 2.0 Hz, 1H), 8.45 (s, 1H), 7.14 (s, 2H), 6.90 (t, *J* = 8.4 Hz, 1H), 6.31 (d, *J* = 10.4 Hz, 2H), 5.41 (s, 2H), 5.29 (s, 2H); ^13^C NMR (100 MHz, DMSO-d_6_) δ 162.4, 156.47 (d, *J*F = 13.9 Hz), 154.52, 152.90, 151.08 (d, *J*F = 11.5 Hz), 139.86, 133.74, 132.78, 131.30 (d, *J*F = 5.7 Hz), 117.12, 110.27, 110.21, 110.04, 105.13, 100.20 (d, *J*F = 23.8 Hz), 43.26 (d, *J*F = 3.3 Hz); HRMS (ESI) calc. for C_18_H_13_FN_8_ [M + H]^+^ 360.1247, found: 360.1251.

##### 4-(6-Amino-1-(4-amino-2-fluorobenzyl)-1*H*-pyrazolo[3,4-*d*]pyrimidin-4-yl)picolinonitrile (**11h**)

Compounds **9b** and **10h** were sequentially processed according to general procedure C to yield **11h** as yellow solid (7% yield, 95.76% purity); ^1^H NMR (400 MHz, DMSO-d_6_) δ 8.97 (d, *J* = 5.2 Hz, 1H), 8.60 (s, 1H), 8.44 (s, 1H), 8.40 (dd, *J* = 5.2, 1.6 Hz, 1H), 7.19 (s, 2H), 6.89 (t, *J* = 8.4 Hz, 1H), 6.30 (d, *J* = 10.8 Hz, 2H), 5.41 (s, 2H), 5.29 (s, 2H); ^13^C NMR (100 MHz, DMSO-d_6_) δ 162.2, 162.0, 159.8, 156.1 (d, *J* = 6.6 Hz, 1C), 152.2, 150.6 (d, *J* = 11.5 Hz, 1C), 145.2, 133.6, 133.1, 130.9, 127.4, 126.4, 117.4, 109.7, 109.6, 104.7, 99.7 (d, *J* = 23.9 Hz, 1C), 42.9 (d, *J* = 3.2 Hz, 1C); HRMS (ESI) calc. for C_18_H_13_FN_8_ [M + H]^+^ 360.1247, found: 360.1244.

##### 3-(6-Amino-1-(4-amino-2-fluorobenzyl)-1*H*-pyrazolo[3,4-*d*]pyrimidin-4-yl)benzonitrile (**11i**)

Compounds **9b** and **10i** were sequentially processed to yield **11i** as off-white solid (19% yield, 97.66% purity); ^1^H NMR (400 MHz, DMSO-d_6_) δ 8.51 (s, 1H), 8.47 (d, *J* = 8.0 Hz, 1H), 8.35 (s, 1H), 8.06 (d, *J* = 7.6 Hz, 1H), 7.79 (t, *J* = 7.8 Hz, 1H), 7.07 (s, 2H), 6.88 (t, *J* = 8.4 Hz, 1H), 6.32–6.29 (m, 2H), 5.40 (s, 2H), 5.28 (s, 2H); ^13^C NMR (100 MHz, DMSO-d_6_) δ 162.1, 162.0, 158.2, 156.0, 150.6, 150.5, 137.7, 134.4, 133.2, 133.1, 131.7, 130.8 (d, *J* = 6.7 Hz, 1C), 130.2, 118.4, 112.1, 109.8 (d, *J* = 12.5 Hz, 1C), 104.4, 99.7 (d, *J* = 24.1 Hz, 1C), 42.8 (d, *J*F = 4.1 Hz, 1C); HRMS (ESI) calc. for C_19_H_14_FN_7_ [M + H]^+^ 359.1295, found: 359.1304.

##### 3-(6-Amino-1-(4-amino-2-fluorobenzyl)-1*H*-pyrazolo[3,4-*d*]pyrimidin-4-yl)-2-fluorobenzonitrile (**11j**)

Compounds 9b and 10j were sequentially processed to yield 11j as white solid (31% yield, 97.40% purity); ^1^H NMR (400 MHz, DMSO-d_6_) δ 8.21–8.10 (m, 2H), 7.98 (d, J = 3.7 Hz, 1H), 7.61 (t, J = 7.8 Hz, 1H), 7.14 (s, 2H), 6.97–6.84 (m, 1H), 6.32 (s, 1H), 6.31–6.28 (m, 1H), 5.42 (s, 2H), 5.27 (s, 2H); ^13^C NMR (100 MHz, DMSO-d_6_) δ 162.16, 161.31, 159.72, 158.71, 155.34, 155.06, 150.60 (d, J = 11.6 Hz), 136.56 (d, JF = 3.3 Hz), 135.84, 133.51 (d, JF = 8.3 Hz), 130.86 (d, JF = 5.8 Hz), 125.88 (d, JF = 4.1 Hz), 125.65 (d, JF = 11.6 Hz), 113.90, 109.82, 109.63, 106.17, 101.51 (d, JF = 15.8 Hz), 99.72 (d, JF = 24.0 Hz), 42.75; HRMS (ESI) calc. for C_19_H_13_F_2_N_7_ [M + H]^+^ 377.1200, found: 377.1210.

##### 2-(6-Amino-1-(4-amino-3-methylbenzyl)-1*H*-pyrazolo[3,4-*d*]pyrimidin-4-yl)isonicotinonitrile (**11k**)

Compounds **9c**, **10k** were sequentially processed to yield **11k** as yellow solid (10% yield, 96.15% purity); ^1^H NMR (400 MHz, DMSO-d_6_) δ 9.08 (dd, *J* = 4.8, 0.8 Hz, 1H), 8.67 (dd, *J* = 1.6, 1.2 Hz, 1H), 8.45 (s, 1H), 8.07 (dd, *J* = 5.2, 1.6 Hz, 1H), 7.10 (s, 2H), 6.86 (s, 1H), 6.82 (dd, *J* = 8.2, 1.8 Hz, 1H), 6.51 (d, *J* = 8.0 Hz, 1H), 5.23 (s, 2H), 4.80 (s, 2H), 1.98 (s, 3H); ^13^C NMR (100 MHz, DMSO-d_6_) δ = 162.0, 156.6, 156.4, 155.7, 151.1, 146.1, 134.8, 129.5, 127.1, 126.1, 124.4, 123.6, 120.9, 120.6, 116.6, 113.7, 104.4, 49.2, 17.4; HRMS (ESI) calc. for C_19_H_16_N_8_ [M + H]^+^ 356.1498, found: 356.1493.

##### 6-(6-Amino-1-(4-amino-3-methylbenzyl)-1*H*-pyrazolo[3,4-*d*]pyrimidin-4-yl)picolinonitrile (**11l**)

Compounds **9c** and **10l** were sequentially processed to yield **11l** as yellow solid (10% yield, 98.87% purity); ^1^H NMR (400 MHz, DMSO-d_6_) δ 8.65 (dd, *J* = 8.0, 0.8 Hz, 1H), 8.39 (s, 1H), 8.31 (t, *J* = 8.0 Hz, 1H), 8.24 (dd, *J* = 7.6, 1.2 Hz, 1H), 7.11 (s, 2H), 6.86 (s, 1H), 6.82 (dd, *J* = 8.2, 1.8 Hz, 1H), 6.52 (d, *J* = 8.0 Hz, 1H), 5.24 (s, 2H), 4.80 (s, 2H), 1.99 (s, 3H); ^13^C NMR (100 MHz, DMSO-d_6_) δ = 162.0, 156.5, 156.3, 155.9, 146.0, 139.4, 134.5, 132.6, 130.7, 129.5, 126.0, 125.5, 124.4, 120.9, 117.3, 113.7, 104.3, 49.2, 17.4; HRMS (ESI) calc. for C_19_H_16_N_8_ [M + H]^+^ 356.1498, found: 356.1488.

##### 5-(6-Amino-1-(4-amino-3-methylbenzyl)-1*H*-pyrazolo[3,4-*d*]pyrimidin-4-yl)nicotinonitrile (**11m**)

Compounds **9c** and **10m** were sequentially processed to yield **11m** as yellow solid (15% yield, 97.57% purity); ^1^H NMR (400 MHz, DMSO-d_6_) δ 9.53 (d, *J* = 2.0 Hz, 1H), 9.21 (d, *J* = 2.0 Hz, 1H), 8.91 (t, *J* = 2.2 Hz, 1H), 8.43 (s, 1H), 7.12 (s, 2H), 6.88 (d, *J* = 1.6 Hz, 1H), 6.83 (dd, *J* = 8.4, 2.0 Hz, 1H), 6.52 (d, *J* = 8.0 Hz, 1H), 5.23 (s, 2H), 4.80 (s, 2H), 1.99 (s, 3H); ^13^C NMR (100 MHz, DMSO-d_6_) δ 162.0, 156.0, 155.8, 154.0, 152.4, 146.1, 139.4, 133.0, 132.4, 129.6, 126.2, 124.3, 120.9, 116.7, 113.7, 109.6, 104.8, 49.2, 17.5; HRMS (ESI) calc. for C_19_H_16_N_8_ [M + H]^+^ 356.1498, found: 356.1495.

##### 4-(6-Amino-1-(4-amino-3-methylbenzyl)-1*H*-pyrazolo[3,4-*d*]pyrimidin-4-yl)picolinonitrile (**11n**)

Compounds **9c** and **10n** were sequentially processed to yield **11n** as yellow solid (7% yield, 95.38% purity); ^1^H NMR (400 MHz, DMSO-d_6_) δ 8.97 (d, *J* = 4.8 Hz, 1H), 8.60 (s, 1H), 8.44 (s, 1H), 8.40 (dd, *J* = 5.0, 1.4 Hz, 1H), 7.17 (s, 2H), 6.88 (s, 1H), 6.83 (d, *J* = 8.4 Hz, 1), 6.52 (d, *J* = 8.0 Hz, 1H), 5.24 (s, 2H), 4.81 (s, 2H), 1.99 (s, 3H); ^13^C NMR (100 MHz, DMSO-d_6_) δ 161.99, 155.98, 155.95, 152.18, 146.09, 145.24, 133.54, 132.79, 129.58, 127.33, 126.37, 126.17, 124.24, 120.86, 117.34, 113.69, 104.80, 49.25; HRMS (ESI) calc. for C_19_H_16_N_8_ [M + H]^+^ 356.1498, found: 356.1493.

##### 2-[6-Amino-1-[(4-amino-2,6-difluoro-phenyl)methyl]pyrazolo[3,4-*d*]pyrimidin-4-yl]pyridine-4-carbonitrile (**11p**)

Compounds **9d** and **10p** were sequentially processed to yield **11p** as yellow solid (16% yield, 98.30% purity); ^1^H NMR (400 MHz, DMSO-d_6_) δ 9.07 (dd, *J* = 4.8, 0.8 Hz, 1H), 8.67 (d, *J* = 0.8 Hz, 1H), 8.41 (s, 1H), 8.06 (dd, *J* = 4.8, 1.6 Hz, 1H), 7.11 (s, 2H), 6.20–6.14 (m, 2H), 5.79 (s, 2H), 5.26 (s, 2H); ^13^C NMR (100 MHz, DMSO-d_6_) δ 161.89, 156.53, 156.42, 155.66, 151.13 (d, *J* = 4.1 Hz, 1C), 135.07, 127.13, 123.61, 120.60, 116.63, 104.13, 97.91, 96.07, 95.79, 36.82 (br s, 1C); HRMS (ESI) calc. for C_18_H_12_F_2_N_8_ [M + H]^+^ 378.1153, found: 378.1155.

##### 6-[6-Amino-1-[(4-amino-2,6-difluoro-phenyl)methyl]pyrazolo[3,4-*d*]pyrimidin-4-yl]pyridine-2-carbonitrile (**11q**)

Compounds 9d and 10q were sequentially processed to yield 11q as yellow solid (21% yield, 95.11% purity); ^1^H NMR (400 MHz, DMSO-d_6_) δ 8.65 (dd, *J* = 7.8, 1.0 Hz, 1H), 8.34 (s, 1H), 8.31 (t, *J* = 7.8 Hz, 1H), 8.23 (dd, *J* = 7.8, 1.0 Hz, 1H), 7.12 (s, 2H), 6.18 (d, *J* = 10.0 Hz, 2H), 5.79 (s, 2H), 5.26 (s, 2H); ^13^C NMR (100 MHz, DMSO-d_6_) δ 163.33, 161.93, 156.48, 156.33, 155.82, 139.44, 134.79, 132.58, 130.66, 125.54, 117.32, 104.09, 97.91, 96.06, 95.79, 36.86; HRMS (ESI) calc. for C_18_H_12_F_2_N_8_ [M + H]^+^ 378.1153, found: 378.1151.

##### 5-[6-Amino-1-[(4-amino-2,6-difluoro-phenyl)methyl]pyrazolo[3,4-*d*]pyrimidin-4-yl]pyridine-3-carbonitrile (**11r**)

Compounds **9d** and **10r** were sequentially processed to yield **11r** as yellow solid (30% yield, 99.42% purity); ^1^H NMR (400 MHz, DMSO-d_6_) δ 9.52 (d, *J* = 2.0 Hz, 1H), 9.21 (d, *J* = 2.0 Hz, 1H), 8.89 (t, *J* = 2.0 Hz, 1H), 8.38 (s, 1H), 7.13 (s, 2H), 6.18 (d, *J* = 10.4 Hz, 2H), 5.25 (s, 2H); ^13^C NMR (100 MHz, DMSO-d_6_) δ 163.75 (d, *J* = 11.5 Hz, 1C), 162.37, 161.33 (d, *J* = 11.5 Hz, 1C), 156.36 (d, *J* = 18.0 Hz, 1C), 154.47, 152.89, 151.64, 139.82, 133.79, 132.80, 117.12, 110.03, 104.98, 98.32, 96.85–95.49 (m, 1C), 37.33 (br s, 1C) ; HRMS (ESI) calc. for C_18_H_12_F_2_N_8_ [M + H]^+^ 378.1153, found: 378.1155.

##### 4-[6-Amino-1-[(4-amino-2,6-difluoro-phenyl)methyl]pyrazolo[3,4-*d*]pyrimidin-4-yl]pyridine-2-carbonitrile (**11s**)

Compounds **9d** and **10s** were sequentially processed to yield **11s** as yellow solid (24% yield, 95.51% purity); ^1^H NMR (400 MHz, DMSO-d_6_) δ 8.97 (dd, *J* = 5.2, 0.8 Hz, 1H), 8.59 (dd, *J* = 1.6, 0.8 Hz, 1H), 8.40–8.38 (m, 2H), 7.19 (s, 2H), 6.22–6.16 (m, 2H), 5.80 (s, 2H), 5.27 (s, 2H); ^13^C NMR (100 MHz, DMSO-d_6_) δ 163.3 (d, *J* = 11.6 Hz, 1C), 162.00, 160.8 (d, *J* = 11.5 Hz, 1C), 156.0, 152.2, 151.2, 145.2, 133.5, 133.1, 127.3, 126.4, 117.3, 104.6, 97.8, 96.0–95.8 (m, 1C), 36.9; HRMS (ESI) calc. for C_18_H_12_F_2_N_8_ [M + H]^+^ 378.1153, found: 378.1155.

##### 3-[6-Amino-1-[(4-amino-2,6-difluoro-phenyl)methyl]pyrazolo[3,4-*d*]pyrimidin-4-yl]benzonitrile (**11t**)

Compounds 9d and 10t were sequentially processed to yield 11t as yellow solid (33% yield, 97.91% purity); ^1^H NMR (400 MHz, DMSO-d_6_) δ 8.49 (t, J = 1.4 Hz, 1H), 8.45 (td, J = 8.0, 1.4 Hz, 1H), 8.29 (s, 1H), 8.05 (td, J = 8.0, 1.4 Hz, 1H), 7.79 (t, J = 7.8 Hz, 1H), 7.07 (s, 2H), 6.21–6.15 (m, 2H), 5.80 (s, 2H), 5.25 (s, 2H); ^13^C NMR (100 MHz, DMSO-d_6_) δ 163.28 (d, J = 12.3 Hz, 1C), 161.94, 160.86 (d, J = 12.3 Hz, 1C), 158.11, 155.93, 151.16, 137.75, 134.36, 133.27, 133.11, 131.74, 130.26, 118.43, 112.13, 104.28, 97.91, 96.15–95.93 (m, 1C), 95.78, 36.84 (br s, 1C); HRMS (ESI) calc. for C_19_H_13_F_2_N_7_ [M + H]^+^ 377.1200, found: 377.1202.

##### 3-(6-Amino-1-(4-amino-2,6-difluorobenzyl)-1*H*-pyrazolo[3,4-*d*]pyrimidin-4-yl)-2-fluorobenzonitrile (**11u**)

Compounds **9d** and **10u** were sequentially processed to yield **11u** as yellow solid (33% yield, 98.28% purity); ^1^H NMR (400 MHz, DMSO-d_6_) δ 8.14 (dd, *J* = 7.8, 6.6 Hz, 2H), 7.93 (d, *J* = 3.6 Hz, 1H), 7.60 (t, *J* = 7.8 Hz, 1H), 7.13 (s, 2H), 6.20–6.15 (m, 2H), 5.80 (s, 2H), 5.24 (s, 2H); ^13^C NMR (100 MHz, DMSO-d_6_) δ 163.34, 162.11, 161.30, 160.92, 158.71, 155.12 (d, *J* = 23.8 Hz, 1C), 151.18, 136.84–136.24 (m, 1C), 135.84, 133.61 (d, *J* = 8.2 Hz, 1C), 125.90 (d, *J* = 4.1 Hz, 1C), 125.67 (d, *J* = 12.3 Hz, 1C), 113.93, 106.03, 101.51 (d, *J* = 15.6 Hz, 1C), 97.85, 96.25–95.98 (m, 1C), 95.87–95.36 (m, 1C), 36.82 (br s, 1C); HRMS (ESI) calc. for C_19_H_12_F_3_N_7_ [M + H]^+^ 395.1106, found: 395.1117.

##### 2-[6-Amino-1-[[4-amino-3-(trifluoromethyl)phenyl]methyl]pyrazolo[3,4-*d*]pyrimidin-4-yl]pyridine-4-carbonitrile (**11v**)

Compounds **9e** and **10v** were sequentially processed to yield **11v** as yellow solid (53% yield, 97.10% purity); ^1^H NMR (400 MHz, DMSO-d_6_) δ 9.08 (dd, *J* = 5.2, 0.8 Hz, 1H), 8.67 (d, *J* = 0.8 Hz, 1H), 8.47 (s, 1H), 8.07 (dd, *J* = 4.8, 1.6 Hz, 1H), 7.28 (d, *J* = 1.6 Hz, 1H), 7.18 (dd, *J* = 8.6, 1.4 Hz, 1H), 7.14 (s, 2H), 6.77 (d, *J* = 8.4 Hz, 1H), 5.59 (s, 2H), 5.31 (s, 2H); ^13^C NMR (100 MHz, DMSO-d_6_) δ 162.0, 156.7, 156.5, 155.6, 151.1, 145.7, 135.2, 132.6, 127.2, 125.3, 123.9, 123.6, 120.6, 117.0, 116.6, 110.1, 110.0, 104.3, 48.4; HRMS (ESI) calc. for C_19_H_13_F_3_N_8_ [M + H]^+^ 410.1215, found: 410.1217.

##### 3-(6-Amino-1-(4-amino-3-(trifluoromethyl)benzyl)-1*H*-pyrazolo[3,4-*d*]pyrimidin-4-yl)benzonitrile (**11w**)

Compounds **9e** and **10w** were sequentially processed to yield **11w** as white solid (21% yield, 99.14% purity).; ^1^H NMR (400 MHz, DMSO-d_6_) δ 8.52 (s, 1H), 8.47 (d, *J* = 8.0 Hz, 1H), 8.38 (s, 1H), 8.06 (d, *J* = 7.6 Hz, 1H), 7.79 (t, *J* = 7.8 Hz, 1H), 7.30 (d, *J* = 1.2 Hz, 1H), 7.21–7.18 (m, 1H), 7.10 (s, 2H), 6.78 (d, *J* = 8.4 Hz, 1H), 5.59 (s, 2H), 5.30 (s, 2H); ^13^C NMR (100 MHz, DMSO-d_6_) δ 162.07, 158.28, 156.03, 145.72, 137.66, 134.41, 133.39, 133.11, 132.68, 131.76, 130.24, 126.31, 125.37 (q, *J*F = 5.5 Hz), 123.86, 118.39, 116.98, 112.14, 110.17 (q, *J*F = 29.0 Hz), 104.48, 48.49; HRMS (ESI) calc. for C_20_H_14_F_3_N_7_ [M + H]^+^ 409.1263, found: 409.1259.

##### 3-(6-Amino-1-(4-amino-3-(trifluoromethyl)benzyl)-1*H*-pyrazolo[3,4-*d*]pyrimidin-4-yl)-2-fluorobenzonitrile (**11x**)

Compounds **9e** and **10x** were sequentially processsed to yield **11x** as off-white solid (15% yield, 98.23% purity); ^1^H NMR (400 MHz, DMSO-d_6_) δ 8.17–8.13 (m, 2H), 8.00 (d, *J* = 3.6 Hz, 1H), 7.60 (t, *J* = 7.8 Hz, 1H), 7.30 (d, *J* = 1.6 Hz, 1H), 7.21–7.19 (m, 1H), 7.15 (s, 2H), 6.78 (d, *J* = 8.4 Hz, 1H), 5.60 (s, 2H), 5.29 (s, 2H); ^13^C NMR (100 MHz, DMSO-d_6_) δ 162.24, 155.28 (d, *J*F = 14.8 Hz, 1C), 145.78, 136.58 (d, *J*F = 3.3 Hz), 135.88, 133.84, 132.74, 126.32, 125.89 (d, *J*F = 4.1 Hz), 125.52, 123.77, 117.02, 113.91, 110.15 (d, *J*F = 29.5 Hz), 106.23, 101.53 (d, *J*F = 16.4 Hz), 48.50; HRMS (ESI) calc. for C_20_H_13_F_4_N_7_ [M + H]^+^ 427.1169, found: 427.1170.

### 3.2. In Vitro Human Adenosine Receptor-Binding and Functional Assays

The radioligand-binding assays for adenosine receptors were performed at Eurofins Discovery Services (Panlabs, Taiwan). Briefly, stock solutions of the compounds were prepared in DMSO and further diluted with the incubation buffer to the desired concentration. Final DMSO concentrations in the assay were 1.0%. For adenosine A_1_ screening, a 10 μg aliquots of membrane preparations from human recombinant CHO-K1 cells stably expressing A_1_ receptors (CHO-K1-hA1AR clone3, EPDST, R200510) were incubated with 1 nM [^3^H]DPCPX in incubation buffer (20 mM HEPES, pH 7.4, 10 mM MgCl_2_, 100 mM NaCl 1.40 nM) for 90 min at 25 °C. Nonspecific binding was estimated in the presence of 100 μM R(-)-PIA. Membranes were filtered and washed 3 times and the filters were counted to determine specifically bound [^3^H]DPCPX. Chemical compounds were screened at 10 μM. For A_2A_ screening, membrane preparations from human recombinant HEK-293 cells stably expressing A_2A_ receptors (PerkinElmer, RBHA2AM) were incubated with 0.05 μM [^3^H]CGS-21680 in incubation buffer (50 mM Tris-HCl, pH 7.4, 10 mM MgCl_2_, 1 mM EDTA, 2 U/mL Adenosine Deaminase) for 90 min at 25 °C. Nonspecific binding was estimated in the presence of 50.0 μM NECA. Membranes were filtered and washed 3 times and the filters were counted to determine [^3^H]CGS-21680 specifically bound. For A_2B_ screening, membrane preparations from human recombinant HEK-293 cells stably expressing A_2B_ receptors (PerkinElmer, ES-013-M) were incubated with 1.6 nM [^3^H]MRS1754 in incubation buffer (50 mM Tris-HCl, pH 6.5, 5 mM MgCl_2_, 1 mM EDTA, 0.01 % Bacitracin) for 90 min at 25 °C. Nonspecific binding was estimated in the presence of 100 μM NECA. Membranes were filtered and washed 3 times and the filters were counted to determine [^3^H]MRS1754 specifically bound. For A_3_ screening, membrane preparations from human recombinant CHO-K1 cells stably expressing A_3_ receptors (PerkinElmer, ES-012-M) were incubated with 0.5 nM [^125^I]AB-MECA in incubation buffer (25 mM HEPES, pH 7.4, 5 mM MgCl_2_, 1 mM CaCl_2_, 0.1 % BSA) for 60 min at 25 °C. Nonspecific binding was estimated in the presence of 1.0 μM IB-MECA.

IC_50_ values were determined by a nonlinear, least-squares regression analysis using MathIQ^TM^ (ID Business Solutions Ltd., Guildford, UK). Where inhibition constants (K_i_) were presented, the K_i_ values were calculated using Cheng and Prusoff equation [18] using the observed IC_50_ of the compound, the concentration of radioligand employed in the assay, and the historical values for the K_D_ of the ligand (obtained experimentally at Eurofins Panlabs, Inc.). The Hill coefficient (n_H_), which defines the slope of the competitive binding curve, was calculated using MathIQ^TM^. Hill coefficients significantly different than 1.0, may suggest that the binding displacement does not follow the laws of mass action with a single binding site.

For functional studies, A_1_ and A_2A_ receptor cyclic AMP HTRF (homogeneous time resolved fluorescence) assay was performed at EuroscreenFast (Charleroi, Belgium) using CHO-K1 (A_1_) and HEK293 (A_2A_) stable cell line with CPA (A_1_) and NECA (A_2A_) as reference agonist and DPCPX (A_1_) and ZM241385 (A_2A_) as reference antagonist compound.

### 3.3. hERG, AMES and Metabolic Stability Study

*h*ERG ion channel-blocking profile of acute exposure to **11o** was performed in stably expressing HEK cells at Nova Research Laboratories (New Orleans, LA, USA). Briefly, cloned equivalent of the human IKr, *h*ERG, was used. Cells were obtained from Cytogentrics Bioscience GmbH (Jachim-Jungius-Strafe 9, 18,059 Rostock, Germany). Internal and external recording solutions as well as data acquisition and analysis were as previously described [15].

AMES bacterial reverse-mutation test was performed at Frontage laboratories inc. (Frontage laboratories, Concord, OH, USA) according to testing facility’s standard operating procedures (SOPs) following OECD guideline 471, “Bacterial Reverse Mutation Test”, and ICH S2(R1), “Guidance on Genotoxicity Testing and Data Interpretation for Pharmaceuticals Intended for Human Use”[19]. Briefly, test system was exposed to test article via plate-incorporation methodology originally described by Ames et al. [16] and updated by Maron and Ames [17]. This test system has been shown to detect a wide range of classes of chemical mutagens [16,20]. For mutagenicity assay, TA98 and TA100 (tester strains source: Molecular Toxicology Inc. (Boone, NC, USA). TA98 lot number 5464D, part number 71–098L; TA100 lot number 5439D, part number 71–100L) was exposed to vehicle alone and at least 8 concentrations of **11o**, with duplicate plates/condition, in both the presence and absence of S9 using the plate incorporation method. Unless limited by solubility, **11o** was to be evaluated at a maximum concentration of 5000 μg/plate (the dose levels were 5000, 1581, 500, 158, 50, 15.8, 5.0 and 1.5 μg/plate). However, **11o** showed limited solubility and the highest tested concentration permissible as a workable preparation was 1867 μg/plate. Following incubation, plates were examined for the condition of the bacterial background lawn and precipitate and recorded. Colonies were enumerated either by visually or by an automatic colony counter (Scan 1200 Automated colony counter and scan software).

Metabolic stability of **11o** was determined in cryopreserved hepatocytes from mice, rats, dogs, monkeys and human according to the SOPs of Frontage Laboratories Inc. Briefly, cryopreserved hepatocytes for pre-clinical animals and for human (BioIVT, Westbury, NY, USA) were thawed in a 37 °C water bath with gentle shaking until ice had almost melted. The suspensions were immediately transferred to a centrifuge tube (50 mL) containing pre-warmed thawing media at 37 °C with gentle handshaking to prevent the cells from settling. Cell suspensions were centrifuged at 50× *g* and 4 °C for 5 min. The supernatant was discarded and the pellets were resuspended in prewarmed incubation media (5 mL) at 37 °C. The percentage of viable cells in the suspension was determined by Trypan blue staining method to ensure viability of ≥70 %. **11o** was incubated in a 24-well plate containing hepatocyte suspensions (1million cells/mL) at 37 °C for 4 h under 5% CO_2_ and 95% air. Aliquots were removed in duplicate at 0, 15, 30, 60, 120, 180 and 240 min and the samples were treated with ACN (3 volumes containing an internal standard). The samples were centrifuged and aliquots of the supernatant were analyzed for parent compounds by LC/MS/MS using suitable multiple reaction monitoring (MRM) transitions. Midazolam (0.2 μM) was included as positive control to confirm the viability of the hepatocytes from each species. The disappearance of **11o** and midazolam was monitored by LC/MS/MS (MRM) analysis of the hepatocyte extracts at each time point. The peak area ratio of the analyte to internal standard was obtained for each sample and the rate of disappearance of **11o** was assessed by comparing the peak area ratios at various time points to the peak area ratio obtained at t = 0. The t_1/2_ is obtained (using the linear portion of semi-log plot of the area ratio vs. time) and the CL_int_ values determined using well-stirred model.

### 3.4. Animal Studies

#### 3.4.1. MPTP-Induced Mouse Model of Parkinson’s Disease

Male C57BL/6J mice of 10 weeks of age were purchased from Charles River Laboratories (Charles River Laboratories, Freiburg, Germany) and maintained at QPS Austria. All procedures described were reviewed and approved by IACUC at QPS Austria. Animal studies conformed to the Austrial guidelines for the care and use of laboratory animals (Tierversuchsgesetz 2012-TVG 2012, BGB1. I Nr. 114/2012). Animal experiments were approved by the Styrian government (Amt der Steiermarkischen Landersregierung, Abteilung 13—Umwelt and Raumordnung Austria; ABT13-78Jo234-2018).

As soon as the animals arrived, they were brought to the assigned animal room, unpacked and checked for their health status. Animals were habituated at least for one week prior to study start. Animals were single-housed in individual ventilated cages on standardized rodent bedding supplied by Rettenmaier. The room temperature was maintained at 20~24 °C and the relative humidity was maintained between 45 to 65%. Animals were housed under a constant light cycle (12 h light/dark). Dried, pelleted standard rodent chow (Altromin) as well as normal tap water was available to the animals ad libitum.

Animals with apparently good health conditions were included to the study. All animals were randomly assigned to starting groups (cohort). The number of animals in a starting group was limited to ensure same age and uniform handling. A total of 144 male C57BL/6J mice were allocated to 12 groups (*n* = 12/group). Eleven groups (groups 2~12) were intraperitoneally (IP) treated four times with 20 mg/kg MPTP with 2 h interval on day 1. Probenecid or vehicle was administered via IP injection (250 mg/kg) with the first MPTP or vehicle treatment (e.g., MPTP left abdomen, Probenecid right abdomen). One group received vehicle only (group 1) and served as control. Group 4 received a positive control/reference item (rasagiline) via IP injection 30 min before the first MPTP injection. Thirty minutes after the last MPTP treatment the first compound treatment was performed. Groups received either vehicle (group 2 PO and 3 IN), four different doses of **11o** via oral gavage (groups 5~8) or intranasal (groups 9~12). Animals were treated with the compound or vehicle for 7 consecutive days. For PO, each mouse received 10 μL/g body weight of compound. For IN, the body weight of animals was averaged to 30 g. This was basis for concentration calculation for the whole treatment period. Each animal received a volume of 5 μL per naris (10 μL per animal).

#### 3.4.2. Nest Building

Behavior assessment was performed on day 3, two hours after the scheduled treatment. All animals were tested in nest-building and open-field testing in a randomized order.

To test individual nest-building behavior, mice were housed individually in cages containing wood-chip bedding and one square of pressed cotton (‘nestlet’). No other nesting material (e.g., wood wool) was present. The nestlet was introduced on the day before the evaluation of the nest status in about 2 to 3 h before the dark phase was initiated, and the nest-building behavior was evaluated on the following day of the experiment within 2~3 h after the light-phase start. Time span between introduction of the cotton square and evaluation of the nest status was same for all examinations. Manipulation of the nestlet and the constitution of the built nest were assessed according to five-point scale [21] as follows: 1. Nestlet not noticeably touched (>90%) intact); 2. Nestlet partially torn up (50%~90% remaining intact); 3. Mostly shredded but often not identifiable nest site: <50% of the nestlet remains intact but <90% is within a quarter of the cage floor area, i.e., the cotton is not gathered into a nest, but spread around the cage; 4. An identifiable, but flat nest: >90% of the nestlet is torn up, the material is gathered into a nest within a quarter of the cage floor area, but the nest is flat, with walls higher than mouse body height (curled up on its side) on less than 50% of its circumference; 5. A (near) perfect nest: >90% of the nestlet is torn up, the nest is a crater, with walls higher than mouse body height on more than 50% of its circumference.

#### 3.4.3. Haloperidol-Induced Catalepsy in Female Rats

A total of 120 female Sprague Dawley rats (275~300 g) were purchased from Charles River Laboratories (Charles River, Senneville, QC, Canada) and maintained at Atuka, Inc. All animal studies were conducted at Atuka, Inc. according to CCAC guidelines and under IACUC-approved Animal Use Protocols (AUPs), with IACUC number 6606.0.3 for this study.

A one-week period of acclimatization was allowed between delivery of rats and commencement of treatments. Animals were weighed on the first day of acclimatization and then on a weekly basis thereafter. Rats were housed 2/cage at standard temperature (21 ± 2 °C) in a light-controlled environment (lights on 6:00 am to 6:00 pm) with access to food (Teklad 7912, Harlan, Madison, WI, USA) and water ad libitum. The studies were conducted according to CCAC guidelines and under IACUC-approved Animal Use Protocols (AUPs).

The catalepsy study incorporated 12 groups of rats. Ten rats were incorporated into each treatment group (total N = 120 rats). Treatments were divided over 3 consecutive treatment blocks and the order of treatments were randomized within each block and all treatments completed within that block before proceeding to the next. Vehicle (0.5% methyl cellulose, Sigma-Aldrich, St-Louis, MI, USA), **11o** or istradefylline was administered PO at t = 0 min and followed 60 min later (t = 60 min) by haloperidol (1 mg/kg, SC). Catalepsy was assessed a further 60 min later (t = 120).

Behavior was evaluated in each animal by assessing performance on the bar test. Catalepsy was assessed 60 min following haloperidol administration (120 min following administration of vehicle, **11o** or istradefylline). To assess catalepsy, animals were positioned to place both front paws upon a dowel rod (approximately 0.7 cm diameter) suspended 6 cm above a stable surface. The time taken for the animal to remove both paws (decent latency) from the rod and place them back on the surface, in seconds was determined. The test was conducted in triplicate and the average of the three tests were calculated.

### 3.5. In Vivo Pharmacokinetics, Bioavailability and Brain Plasma Ratio Study in Mouse

Male Crl:CD-1 mice of 9~10 weeks of age were purchased from Charles River Laboratories, USA and maintained at standard food and water ad libitum in temperature-controlled room with 12 h on-off light cycle before the experiment. All procedures described were reviewed and approved by the Institutional Animal Care and Use Committee (IACUC) at Frontage Laboratories Inc.

For PO dosing, mice were given **11o** dissolved in DMSO: PEG400 (1:3, *v*:*v*) via gavage. For IV dosing, **11o** solubilized in DMSO: PEG400: saline (1:3:6, *v*:*v*) were injected into tail veins at dose volume of 1 mL/kg. For IN dosing, **11o** solubilized in DMSO and 25 μL of the appropriate dosing solution was placed near the nares to allow animal to inhale the dose. Three mice were sampled from each group for each blood and brain collection time point. Time points are 0.5, 1, 2, 4, 8, 24 h for PO and IN dosing and 0.083, 1, 2, 4, 8 and 24 h for IV dosing. Mice were euthanized by terminal retro-orbital blood collection under isoflurane anesthesia, followed by opening of the thoracic cavity per facility SOPs. A target volume of 1 mL was collected into tubes containing K_3_EDTA anti-coagulant, inverted several times and held on wet ice until centrifuged under refrigeration (set at 5 °C for 10 min at 2000× *g*). Resulting plasma were stored in a freezer set to maintain −70 °C until analyzed for **11o** concentration. Brain was removed from each animal and weighed following termination, snap frozen and stored until later use.

Plasma and brain PK analysis was conducted using WinNolin Version 6.2.1 (Pharsight, Mountain View, CA, USA), operating as a validated software system. Noncompartmental analysis was conducted using the extravascular administration model for oral dosing and the IV bolus model for IV dosing. The peak plasma and brain concentration, time to achieve peak plasma concentration, half-life, and area under the plasma concentration-time curve (C_max_, T_max_, T_1/2_ and AUC) were calculated from mean plasma concentrations for each sampling time/dose group for **11o**. Nominal blood collection times were used for calculation of PK parameters. Dose proportionality was assessed, and bioavailability was calculated for PO dosing.

### 3.6. Maximum Tolerated Dose Study in Rat and Dog

Rat and dog MTD studies were performed at Frontage Laboratories Inc following testing facility’s SOPs. All procedures described were reviewed and approved by the Institutional Animal Care and Use Committee (IACUC) at Frontage Laboratories Inc.

For rat MTD study, male and female Sprague Dawley rats (*Rattus norvegicus*) of 8.1~9.6 weeks of age were purchased from Charles River Laboratories, USA and maintained at standard food and water ad libitum in temperature-controlled room with 12 h on-off light cycle before the experiment. Animals were acclimated to the testing facility for at least 6 to 16 days prior to initiation of dosing. Animals received a single dose of **11o** in 0.5% methyl cellulose via oral gavage starting from 150 mg/kg dose level (this initial dose level was selected based on available data and the estimated toxicity using EPA’s toxicity estimation software tool (TEST) version 4.2.1) in 10 mL/kg dose volume. Subsequent doses occurred 3 or 4 days following the previous dose and were increased based on findings from the previous dosing(s). The procedure continued until four dosing events occurred. Animals were observed once in the morning and once in the afternoon throughout the study for clinical observations. Body weights were measured on the day prior to dose administration. Data were collected and reported electronically using Provantis^TM^, (Instem LSS Ltd. Staffordshire, UK).

For dog MTD study, male and female beagle dogs (*Canis familiaris*) of 1.3 to 1.4 years of age were from Frontage Laboratories’ test facility stock colony, originally procured from Marshall BioResources (North Rose, NY, USA). The health status of the animals was reviewed by a staff veterinarian. Data collected on animals prior to release to study were maintained as testing facility records. Same dogs were used for each dose event. Animals received a single dose of **11o** in 0.5% methyl cellulose via oral gavage starting from 50 mg/kg dose level in 5 mL/kg dose volume. Subsequent doses occurred 3 or 4 days following the previous dose and were increased based on findings from the previous dosing(s). The procedure continued until four dosing events occurred. Animals were fasted overnight prior to each dose administration with food provided approximately 4 h post-dose. Animals were observed once in the morning and once in the afternoon throughout the study for clinical observations. Body weights were measured on the day prior to dose administration. Data were collected and reported electronically using Provantis^TM^ (Instem LSS Ltd., Staffordshire, UK).

### 3.7. Statistical Analysis

Statistical analysis was performed using GraphPad Prism 8.3.0. Data were tested for normality using the Kolmogorov–Smirnov test. If normal distribution was confirmed, differences between groups were tested with the one-way ANOVA followed by either Bonferroni or Dunnett’s post hoc analysis. If data were not normally distributed, differences between groups were tested with Kruskal–Wallis test, followed by Dunn’s post hoc test for multiple comparisons. Data were presented as mean ± SEM.

## 4. Conclusions

In this study, a series of novel adenosine A_2A_ receptor antagonists with 1*H*-pyrazolo [3,4*-d*]pyrimidin-6-amine core scaffolds was designed and synthesized as potential anti-Parkinson’s disease agents using efficient synthetic methods with three-step short routes. Among them, compound **11o** with a 2-fluoro-3-cyanophenyl **A** group exhibited high selectivity for A_2A_ and A_1_ receptors with full antagonism for both A_1_ and A_2A_ receptors. Animal studies indicated that this compound displayed a positive tendency toward improving nesting behavior in MPTP-induced mouse model of Parkinson’s disease, and a dose-dependent reversal of catalepsy induced by haloperidol in female rats. In vitro metabolism study in hepatocytes from mice, rats, dogs, monkeys and humans showed that the compound exhibited relatively low clearance in these species. When administered orally and intranasally to mice, **11o** exhibited excellent bioavailability, good brain/plasma ratio and PK profile. Compound **11o** had low to no cardiotoxicity risk and mutagenic potential, with MTD in rats and dogs of >1000 mg/kg and >400 mg/kg, respectively. Taken together, these results indicate that **11o** is a dual A_2A_/A_1_ receptor antagonist, and a good potential drug candidate for treating Parkinson’s disease that merits further exploration.

## Figures and Tables

**Table 1:** Binding affinity of synthesized compounds for *h*A_2A_ receptors.

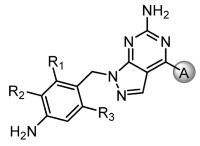						
	Compounds	R_1_	R_2_	R_3_	A_2A_ K_i_ (nM) *	A_1_ K_i_ (nM) *
	Istradefylline				8.64	610
**11a**	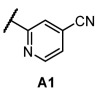	H	H	H	310	2470
**11e**		F	H	H	120	710
**11k**		H	Me	H	65	510
**11p**		F	H	F	47	380
**11v**		H	CF_3_	H	10.2	250
**11b**	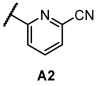	H	H	H	31	650
**11f**		F	H	H	10.6	160
**11l**		H	Me	H	6.39	180
**11q**		F	H	F	6.84	150
**11c**	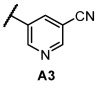	H	H	H	4790	32,000
**11g**		F	H	H	2640	32,800
**11m**		H	Me	H	2480	21,200
**11r**		F	H	F	1220	10,000
**11d**	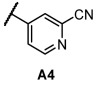	H	H	H	4720	8530
**11h**		F	H	H	3190	28,000
**11n**		H	Me	H	1150	21,300
**11s**		F	H	F	4920	-
**4a**	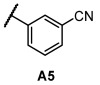	H	H	H	16.38	-
**11i**		F	H	H	12.1	160
**8**		H	Me	H	11.1	110
**11t**		F	H	F	7.42	50
**11w**		H	CF_3_	H	1.94	49
**4b**	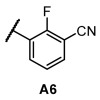	H	H	H	19.4	-
**11j**		F	H	H	16.4	63
**11o**		H	Me	H	13.3	55
**11u**		F	H	F	6.45	30
**11x**		H	CF_3_	H	2.71	33

* Value is the average of the duplicate experiments.

**Table 2:** Selectivity of compound **11o** toward human adenosine receptors based on its binding activities.

Adenosine Receptors	IC_50_ (nM) ^a^	K_i_ (nM) ^a^	n_H_^b^
A_1_	94	55	1.17
A_2A_	24	13.3	0.79
A_2B_	1220	400	0.98
A_3_	1130	1050	0.44

^a^ Value is the average of the duplicate experiments; ^b^ n_H_, Hill coefficient. Values significantly greater or less than 1 represent either positive or negative cooperative binding, respectively, whereas a Hill coefficient of 1 indicates that the affinity of the receptor for the ligand is independent of other bound ligand(s).

**Table 3:** Summary of the half-life, intrinsic clearance and extrapolated clearance values of **11o** and midazolam in mouse, rat, dog, monkey and human hepatocytes.

Species	Half-Life (min)		Intrinsic Clearance (mL/min/10^6^ Cells)		Extrapolated Clearance, CL (mL/min/kg)
	11o	Midazolam ^b^	11o	Midazolam	11o
Mouse	70.9	49.9	0.00977	0.0139	115
Rat	46.9	21.9	0.0148	0.0317	69.2
Dog	ND ^a^	32.8	ND ^a^	0.0211	ND ^a^
Monkey	124	36.0	0.00558	0.0192	20.1
Human	184	68.3	0.00377	0.0101	9.58

^a^ ND: Not determined because little/no compound depletion was observed. ^b^ Midazolam (0.2 μM) was used as the positive control.

**Table 4:** Pharmacokinetic parameters and bioavailability of **11o** in mouse plasma.

Dose (mg/kg)	Dose Route	T_max_ (h)	T_1/2_ (h)	C_max_ (ng/mL)	AUC_last_ (h·ng/mL)	BA ^c^ (%)
1	PO	2.00	4.38	36.8	176	53.0
3	PO	2.00	3.66	81.3	577	57.9
10	PO	2.00	5.69	367	2249	67.7
1	IN	0.50	NC ^a^	23.3	74.9	22.6
3	IN	0.50	NR ^b^	53.9	183	18.4
10	IN	0.50	7.40	88.5	444	13.4
1	IV	0.083	3.95	459	332	100

^a^ NC = not reported due to insufficient data points for the elimination phase or no exposure. ^b^ NR = not reported due to a poor goodness of fit (R_2_ < 0.8) for the elimination phase. ^c^ % Bioavailability (BA) = [(AUC/Dose)/(AUC_IV_/Dose_IV_)] × 100; AUC_last_ for the 1 mg/kg IV dose was used for the calculation.

**Table 5:** Pharmacokinetic parameters and brain/plasma ratio of **11o** in mouse brain.

Dose (mg/kg)	Dose Route	T_max_ (h)	T_1/2_ (h)	C_max_ (ng/mL)	AUC_last_ (h·ng/mL)	B/P Ratio ^c^
1	PO	2.00	NC ^a^	5.58	4.26	0.0242
3	PO	0.50	3.23	12.8	56.4	0.0977
10	PO	2.00	7.71	48.1	375	0.167
1	IN	0.50	NC	9.63	5.23	0.0698
3	IN	0.50	NC	15.9	14.2	0.0776
10	IN	0.50	3.74	38.2	81.3	0.183
1	IV	0.083	NR ^b^	410	246	0.741

^a^ NC = not reported due to insufficient data points for the elimination phase or no exposure. ^b^ NR = not reported due to a poor goodness of fit (R2 < 0.8) for the elimination phase. ^c^ B/P ratio = (Brain AUC_last_/Plasma AUC_last_).

**Table 6:** *h*ERG inhibition, mutagenicity and maximum tolerated dose of **11o**.

Toxicity	
*h*ERG inhibition	IC_50_ = 116 μM
AMES	no mutagenicity up to 1867 μg/plate
MTD (rat)	>1000 mg/kg
MTD (dog)	>400 mg/kg

## Data Availability

Data is contained within the article.
